# Wnt4 is crucial for cardiac repair by regulating mesenchymal-endothelial transition via the phospho-JNK/JNK

**DOI:** 10.7150/thno.71392

**Published:** 2022-05-13

**Authors:** Wenyan Dong, Yue Zhao, Daqiang Wen, Yingjiong Lin, Chui Zeng, Jingkai Gu, Fan Liao, Ruiqi Li, Xu Zhang, Dianliang Wang, Wenqian Cai, Jinzhu Duan

**Affiliations:** 1 Heart Center and Institute of Pediatrics, Guangzhou Women and Children's Medical Center, Guangzhou Medical University, Guangzhou 510120, China; 2 Medical College of South China University of Technology, Guangzhou 510120, China; 3 Stem Cell and Tissue Engineering Research Laboratory, PLA Rocket Force Characteristic Medical Center, Beijing 100088, China

**Keywords:** Cardiac ischemic reperfusion injury, Wnt4, cardiac fibroblast, mesenchymal-endothelial transition (MEndoT), p53

## Abstract

**Rational:** Wnt4 plays a critical role in development and is reactivated during fibrotic injury; however, the role of Wnt4 in cardiac repair remains unclear. In this study, our aim was to clarify the pathophysiological role and mechanisms of Wnt4 following acute cardiac ischemic reperfusion injury.

**Methods and results:** We investigated the spatio-temporal expression of Wnt4 following acute cardiac ischemic reperfusion injury and found that Wnt4 was upregulated as an early injury response gene in cardiac fibroblasts near the injury border zone and associated with mesenchymal-endothelial transition (MEndoT), a beneficial process for revascularizing the damaged myocardium in cardiac repair. Using ChIP assay and *in vitro* and *in vivo* loss- and gain-of-function, we demonstrated that Wnt4 served as a crucial downstream target gene of p53 during MEndoT. Wnt4 knockdown in cardiac fibroblasts led to decreased MEndoT and worsened cardiac function. Conversely, Wnt4 overexpression in cardiac fibroblasts induced MEndoT in these cells via the phospho-JNK/JNK signaling pathway; however, both the p53 and Wnt4 protein levels were dependent on the β-catenin signaling pathway. JNK activation plays a critical role in the induction of MEndoT and is crucial for Wnt4 regulated MEndoT. Moreover, Wnt4 overexpression specifically in cardiac fibroblasts rescued the cardiac function worsening due to genetic p53 deletion by decreasing fibrosis and increasing MEndoT and vascular density.

**Conclusion:** Our study revealed that Wnt4 plays a pivotal role in cardiac repair with involvement of phospho-JNK mediated MEndoT and is a crucial gene for cardiac fibroblast-targeted therapy in heart disease.

## Introduction

Heart disease is still a leading cause of morbidity and mortality worldwide. Activated cardiac fibroblasts play crucial roles during cardiac remodeling and repair following cardiac injury 1,2. Determining the mechanism underlying the regulatory role played by cardiac fibroblasts in cardiac repair is vital for cardiac fibroblast-targeted therapy. Studies performed in the past two decades have shown the plasticity of cardiac fibroblasts, which can be regulated into other cell types for cardiac repair, such as induced pluripotent stem cells 3,4, endothelial cells 5, and cardiomyocytes 6-8. Mesenchymal-endothelial transition (MEndoT) has been shown to be beneficial for cardiac repair and may represent a new strategy for treating heart disease by converting cardiac fibroblasts into endothelial phenotype 9-13. Previous research has shown that MEndoT partly depends on the p53 signaling pathway 12; however, in-depth information is not available regarding the molecular mechanism underlying MEndoT.

The Wnt family comprises 19 lipophilic glycoproteins in mammals and the Wnt signaling pathway can be divided into the canonical or Wnt/β-catenin dependent pathway and the non-canonical or β-catenin-independent pathway 14; these glycoproteins are not only developmentally important in cardiogenesis 15 but also play a critical role in wound repair and regeneration 16-22. To determine the pathophysiological role of specific Wnts in cardiac repair, it is important to analyze the regulation of the Wnt system during this repair. In our previous studies, we analyzed the expression of 19 Wnts in the whole heart; three Wnts, that is, Wnt1, Wnt7a, and Wnt4, were found to be dynamically upregulated after acute cardiac ischemic reperfusion (I/R) injury 2. Wnt1, a canonical Wnt and a neural crest cell marker 23,24, has been found to activate the epicardium and cardiac fibroblasts through β-catenin signaling pathway after acute I/R injury and is important for preserving cardiac function after acute cardiac injury 2. However, the role of the noncanonical Wnt4 had not been investigated further.

Wnt4 plays a critical role in skin, gonadal, renal, and cardiac development 25-28. Moreover, it is reactivated during fibrotic injury, such as that related to cardiovascular disease, chronic kidney fibrosis, and skin wounds 19,29-31. Conditional deletion of Wnt4 in interstitial cells shows that Wnt4 is dispensable for medullary kidney myofibroblast proliferation and differentiation 32. Wnt4 null skin exhibits dermal fibroplasia and upregulated Wnt4 inhibit the TGF-β1-induced transdifferentiation of fibroblasts into myofibroblasts, which suggests that Wnt4 has an antifibrotic effect 33,34. Studies have shown that mesenchymal stem cells can improve cardiac function after cardiac injury by regulating endothelial cells via the Wnt4/β-catenin signaling pathway 35,36. These studies indicate that Wnt4 upregulation may play a beneficial role in cardiac repair; however, the role of Wnt4 itself in cardiac repair remains unclear.

Here, aiming to clarify the pathophyiological role of Wnt4 in cardiac repair, we investigated the spatio-temporal expression of Wnt4 following acute I/R injury and found that it may mainly play role in MEndoT-associated cardiac fibroblasts. Thus, this study provides new insights into the mechanism underlying MEndoT and the role played by Wnt4 in cardiac repair.

## Methods

### Animals

All animal studies were approved by the Institutional Animal Care and Use Committee of the Guangzhou Medical University (Approval number: 2016-025) and the animal experimental procedures conformed to the National Institutes of Health (NIH) guidelines. All mice were of a C57BL/6 background, and the Col1a2-CreERT: R26R^tdTomato^ mice and Col1a2-CreERT: p53^fl/fl^ (Col1a2-CreERT: p53 CKO) mice were reported previously 10,12. Tamoxifen (1 mg, Sigma-Aldrich, St. Louis, MO, USA) was administrated intraperitoneally (IP) at 1 mg·day^-1^ for 10 days to induce Cre-mediated recombination in Col1a2-CreERT mice. Five days after the cessation of tamoxifen, the animals were subjected to acute ischemic reperfusion injury.

### Murine acute ischemic reperfusion injury

Mice are anesthetized and maintained with 1.5% isoflurane and intubated with plastic catheter, which was connected to a volume-cycled ventilator supplying supplemental oxygen at a tide volume of 225-250 mL and respiratory rate of 120-130 strokes/min. Under direct visualization, the pericardial sac is opened and the left anterior descending (LAD) artery is temporally occluded close to its origin with 8-0 suture. Myocardial ischemia is confirmed by myocardial blanching as well as by ST elevation on continuous ECG monitoring. Following 30 min of ischemia, the suture is released to induce reperfusion injury which is confirmed by decreased ST segment elevation on ECG. For sham injury, an identical procedure is followed and a ligature is passed under the LAD without occluding it. The surgeon was blinded to treatment.

### Isolation of cardiac fibroblasts

Briefly, the heart was excised from adult mouse hearts, minced, and sequentially digested in a solution containing 50 U/mL collagenase II and 0.1% trypsin at 37 ºC. The cells from the digestion were pooled and centrifuged after filtered through 40 µm nylon cell strainer. Then, the cells were suspended in DMEM supplemented with 10% fetal bovine serum (FBS), penicillin/streptomycin (100 U/mL),10 ng/mL leukaemia inhibitory factor (LIF) and 10 ng/mL basic fibroblast growth factor (bFGF), and was seeded into culture dishes (100 mm) for 90 min to allow for the preferential attachment of fibroblasts. Further analysis of isolated fibroblasts confirmed no expression of endothelial cell marker CD31.

### Flow Cytometry

The cardiac fibroblasts were harvested using Accutase (Millipore, Billerica, MA, USA) and resuspended in flow cytometric staining buffer (1% FBS + 0.1% NaN_3_ in PBS) without fixation. Flow cytometric analysis of endothelial cell surface marker was performed using CD31-Brilliant Violet 421 (102423, Biolegend, San Diego, CA, USA) antibody by a Beckman-Coulter (Dako) CytoFLEX S. Data was analyzed using FloJo software.

### Viral constructs and transduction

Cardiac fibroblasts were transduced with the pCLenti-U6-Scramble-shRNA-CMV- Puro-WPRE virus, pCLenti-U6-Wnt4-shRNA-CMV-Puro-WPRE virus (Wnt4 gene ID: 22417; sh-Wnt4: 5'- CAGATGTGCAAACGGAACCTT -3', Multiplicity of infection, MOI = 80), pSLenti-CMV-3xFLAG-PGK-Puro-WPRE virus, pSLenti-CMV-Wnt4-3xFLAG-PGK -Puro-WPRE virus (MOI = 40, 60, 80), respectively, after 48 h, cells were collected for gene expression analysis. For knock down of JNK1 and JNK2, cardiac fibroblasts were transduced with the pCLenti-U6-Scramble-shRNA-CMV-EGFP-F2A-Puro-WPRE virus, pCLenti-U6-JNK1-shRNA-CMV-EGFP-F2A-Puro-WPRE virus (sh-JNK1: 5'-GATTTGGAGGAACGAACTAAG-3', MOI = 80), pCLenti-U6-Scramble-shRNA-CMV-mCherry-F2A-Puro-WPRE virus and pCLenti-U6-JNK2-shRNA-CMV-mCherry-F2A-Puro-WPRE virus (sh-JNK2: 5'- CAGACCAAAGTACCCTGGAAT-3', MOI = 80), respectively, after 72 h, cells were collected for gene expression analysis.

For Cre-dependent expression of target gene by AAV virus administration in transgenic mice 37, the shRNA coding sequence targeting mouse Wnt4 (5'-CAGATGTGCAAACGGAACCTT-3') was cloned into the pAAV-CGB-DIO-EGFP-miR30shRNA-WPRE vector, the Wnt4 RNA coding sequence was cloned into the pAAV-CMV-DIO-EGFP-RNA-3xFLAG-WPRE vector. The pAAV-CGB-DIO-Wnt4-miR30shRNA-WPRE virus and pAAV-CGB-DIO-EGFP-Scramble-miR30shRNA-WPRE virus were administrated into the heart of Col1a2-CreERT: R26R^tdTomato^ mice from jugular vein (1 × 10^11^ U per mice resuspended in 100 μL 1 × PBS). The pAAV-CMV-DIO-Wnt4-3xFLAG-WPRE virus and pAAV-CMV-DIO-EGFP-3xFLAG-WPRE virus were administrated into heart of Col1a2-CreERT: p53CKO mice from jugular vein (1 × 10^11^ U per mice resuspended in 100 μL 1 × PBS). After at least 2 weeks, cardiac fibroblasts were isolated and collected for gene expression analysis. All viruses used in our project were produced by Obio Technology (Shanghai, China).

### Singleplex Immunofluorescence staining

Mice were anaesthetized using 2.0% isoflurane after connecting to a ventilator. The left ventricle was perfused with phosphate buffered saline (PBS) and paraformaldehyde (PFA), followed by further fixed for 4 h in 2% PFA at 4 ºC, and then immersed in 30% sucrose solution overnight for cryoprotection before freezing in optimal cutting temperature (O.C.T.) solution. Immunofluorescence staining of frozen sections (7 µm) was performed using primary antibody to Wnt4 (ab91226, 1:100, Abcam, Cambridge, UK), CD31 (ab56299, 1:100, Abcam, Cambridge, UK), cTnI (ab56357, 1:100, Abcam, Cambridge, UK), CD11b (46512S, 1:100, Cell signaling technology, Boston, MA, USA) and relevant fluorescein-conjugated secondary antibodies, following the manufacturer instructions. Labeled sections were probed, and images were captured using a Leica TCS SP8 confocal microscope. Then, 8-10 fields were randomly chosen from each section of the heart and images were captured. The co-localization analysis of confocal images was performed using the ImageJ software (NIH).

### Multiplexed Immunofluorescence staining

The multiplexed immunofluorescence staining 38 was done by using the Four-color anti-rabbit manual IHC kit (0001100020, PANOVUE, Beijing, China) following the manufacturer instructions. In brief, immunofluorescence staining of vascular endothelial cadherin (VECAD) (ab33168, 1:500, Abcam, Cambridge, UK), Wnt4 and tdTomato (LS-C340696, 1:100, LifeSpan BioSciences, Seattle, WA, USA) was sequentially performed by applying primary antibody and the rabbit superpicture polymer detection HRP kit. Using this IHC Kit, three primary antibodies were sequentially visualized in a single TSA (Tyramide signal amplification) slide.

### Chromatin immunoprecipitation (ChIP)

Cardiac fibroblasts (3 × 10^7^) were fixed in 1% formaldehyde and lysed in buffer (50 mM Tris-HCl pH 8, 10 mM EDTA, 1% SDS, protease inhibitor cocktail Set I (539131, Merck Millipore, Billerica, MA, USA)) and sonicated using a epishear multi-sample sonicator, leading to fragments between 300 and 1,000 bp. ChIP was performed using a commercially available ChIP-IT High Sensitivity Kit (53040, Active Motif, Fullerton, CA, USA) according to manufacturer's instructions. DNA-bound protein was immunoprecipitated using an anti-p53 antibody (ab246550, Abcam, Cambridge, UK) and anti-IgG (sc-2025, Santa Cruz, Santa Cruz, CA, USA) as a negative control. The DNA recovered was sequencing and analysed by quantitative polymerase chain reaction (qPCR) to amplified the promoter region of Wnt4: forward 5'-CGCCCGCCTCCGCCGCCAC-3', reverse 5'-CGCAGGGACCGCAGGCACGAA-3', Primers for p53 binding were designed using the SABiosciences'proprietary database (DECipherment of DNA Elements). PCR was performed with equal amounts of specific antibody immunoprecipitated sample, control (IgG) and input. Values were normalized to input measurements and enrichment was calculated using the comparative D-DCt (DDCt) method.

### Real-time quantitative PCR

Total RNA from cultured cardiac fibroblasts was isolated with EZ-press RNA Purification Kit (B0004D, EZBioscience, Roseville, MN, USA) and cDNA was synthesized by using HiScript III RT SuperMix kit (Q711-1, Vazyme, Nanjing, China). qPCR was performed with the 2 × ChamQ Universal SYBR qPCR Master Mix (Q711-2, Vazyme, Nanjing, China) using an QuantStudio^™^6 Flex (ABI, USA). Oligonucleotide primers used are shown in **Table S1**. Fold-changes in gene expression were calculated using the 2^^(-△△Ct)^ method after normalizing to glyceraldehyde 3-phosphate dehydrogenase (GAPDH).

### MEndoT *in vitro* Model and Cell treatment

Serum starved or unstarved cells were cultured in DMEM, 1 × penicillin/streptomycin or DMEM, 1 × penicillin/streptomycin, 10% FBS (Gibco, USA), respectively. Pifithrin-α 50 µM (P4359, Sigma, St. Louis, MO, USA), reactivation of p53 and induction of tumor apoptosis (RITA) 50 µM (S2781, Selleck Chemical, Houston, TX, USA), SP600125 50 µM (S1460, Selleck Chemical) and IWR-1-endo 50 µM (S7086, Selleck Chemical) were added to the cells cultured with the above culture medium. Detecting gene expression and cell function after treatment for 48 h. The recombinant human Wnt4 protein (RPL817Hu01, Cloud-Clone Corp, Houston, USA) was used for Wnt4 overexpression in cardiac fibroblasts with the above culture medium. For activation of JNK, cardiac fibroblasts were treated with recombinant mouse Mitogen Activated Protein Kinase Kinase 7 (MAP2K7) (80 ng/mL, RPD560Mu01, Cloud-Clone Corp, USA) and anisomycin (25 nM, S7409, Selleck, USA).

### Matrigel tube formation and acetylated low-density lipoprotein (Ac-LDL) uptake

Cardiac fibroblasts (6 × 10^4^ per cm^2^) were seeded in growth factor reduced Matrigel basement membrane matrix (354230, BD Biosciences, Franklin Lakes, NJ, USA) coated wells of Nunc Lab-Tek II chamber slides (154534, Thermo Fisher, Waltham, MA, USA) and were treated in serum starved and unstarved cells culture medium overnight at 37 °C, in the presence of 5% CO_2_. For Ac-LDL uptake, cardiac fibroblasts were seeded in 24 well dishs and treated overnight in serum starved and unstarved culture medium, then 30 ug/mL DiI-Ac-LDL (H7970, Sorlarbio, Beijing, China) were added and incubated for 4 h at 37 ºC before imaging. The images were captured using confocal microscope. Tube length and co-localization were analyzed by using image J software.

### Western blotting

Proteins were extracted from cardiac fibroblasts homogenized in an RIPA Lysis containing protease inhibitor (HY-K0010, MedChemExpress, NJ, USA) and phosphatase inhibitor (HY-K0021, MedChemExpress, NJ, USA) and PMSF (ST505, Beyotime, Shanghai, China). Cell lysates were centrifuged at 12,000 g for 10 min at 4 °C. The supernatant was collected, and protein concentration was measured with BCA protein assay kit (P0011, Beyotime, Shanghai, China). For each sample, 15 μg total proteins were loaded for western blot and visualized and detected by Clarity Western ECL Substrate (1705061, Bio-Rad, Hercules, CA, USA). The western blot results were quantified by densitometric analysis using the Image Lab image analyzer (Bio-Rad, Hercules, CA, USA). The primary antibody used in this part were as follow: anti-Wnt4 (dilution 1:1000; ab262696, Abcam), anti-p53 (dilution 1:1000; ab246550, Abcam), anti-VECAD (dilution 1:1000; ab33168, Abcam), anti-Occludin (dilution 1:250; sc-133256, Santa cruz), anti-c-Jun amino-terminal kinase (JNK) (dilution 1:1000; 9252s, Cell signaling technology, Boston, MA, USA), anti-c-Jun amino-terminal kinase 1 (JNK1) (dilution 1:1000; ab199380, Abcam), anti-c-Jun amino-terminal kinase 2 (JNK2) (dilution 1:1000; ab76125, Abcam), anti-phospho-JNK (p-JNK) (dilution 1:1000; 9255s, Cell signaling technology), anti-β-catenin (dilution 1:1000; 9581s, Cell signaling technology), anti-Non-phospho(active) β-catenin (Ser33/37/Thr41) (dilution 1:1000; 8814S, Cell signaling technology), anti-GAPDH (dilution 1:3000; sc-32233, Santa cruz).

### Echocardiography

The echocardiography was conducted using a VisualSonics Vevo 2100 machine and was performed in conscious mice as previous described 2,10. In brief, the hair was removed from the chest of each mouse using depilation cream and then it was gently restrained and placed on a platform followed by rapid echocardiography (< 5 min). Distress was minimized by performing the echocardiography rapidly. Parasternal long axis M-mode images were recorded, and measurements and analysis were then performed as described. The echocardiographer was blinded to the genotype and treatment of the animal being examined.

### Fibrosis assay of histopathology

Heart tissues were sliced into three pieces, including near apex, between mid-ventricle and apex and mid-ventricle, and fixed in 4% PFA for at least 24 h. Fixed tissues were dehydrated, embedded in paraffin and cut into 5 μm sections. Masson's trichrome staining was performed on de-paraffinized sections using standard methods. Percent of fibrosis area was calculated from whole heart sections from three parts using ImageJ software.

### Evans blue and Triphenyltetrazolium chloride (TTC) staining

Combined Evans blue and TTC staining was performed to determine area at risk and infarct area. In brief, after 30 min of ischemia and reperfusion for 24 h, the mice were subjected to thoracotomy and re-ligation of the LAD artery. To demarcate the area at risk, 3% Evans blue solution was perfused into the aorta and coronary arteries. The infusion was discontinued as soon as epicardial blush was observed. Excess Evans Blue was washed off and cut into 1-mm-thick cross sections. The slices were incubated with a 2% TTC solution at 37°C for 15 min to evaluate infarct area/area at risk followed by fixation with PFA. After 15 min, the sections were washed lightly and photographs of each heart section were taken with a microscope. Analysis was done with Image J software.

### Terminal deoxynucleotidyl transferase dUTP nick end fluorescent labelling (TUNEL) staining

Apoptosis in paraffin-embedded heart tissues was assayed by TUNEL staining using the TMR (Red) TUNEL Cell Apoptosis Detection Kit (G1502, Servicebio, China). Cardiomyocytes were identified by cTnI staining and nuclei were stained with DAPI. The number of TUNEL-positive cardiomyocyte and total cardiomyocyte were counted.

### Vascular density

The slides were deparaffinized in xylene and rehydrated in graded ethanol. Antigen retrieval was performed in citrate buffer (citrate pH 6.0) using microwave heating (MWT). Primary antibody to isolectin B4 (B-1205, Vector Labs, Burlingame, CA, USA) were incubated overnight at 4 ºC, and associated fluorescein-conjugated secondary antibodies.

### Statistical Analysis

Statistical analysis was performed using the GraphPad software (Prism). Student's t-test (two-tailed), one-way analysis of variance (ANOVA) with Tukey post-hoc test analysis as appropriate, two-way analysis of variance (ANOVA) were performed as deemed appropriate. A value of *p* < 0.05 was considered statistically significant and the mean ± standard error of the mean (S.E.M.) was presented graphically.

## Results

### Wnt4 was upregulated in MEndoT-associated cardiac fibroblasts after acute cardiac I/R injury

**Figure 1:**
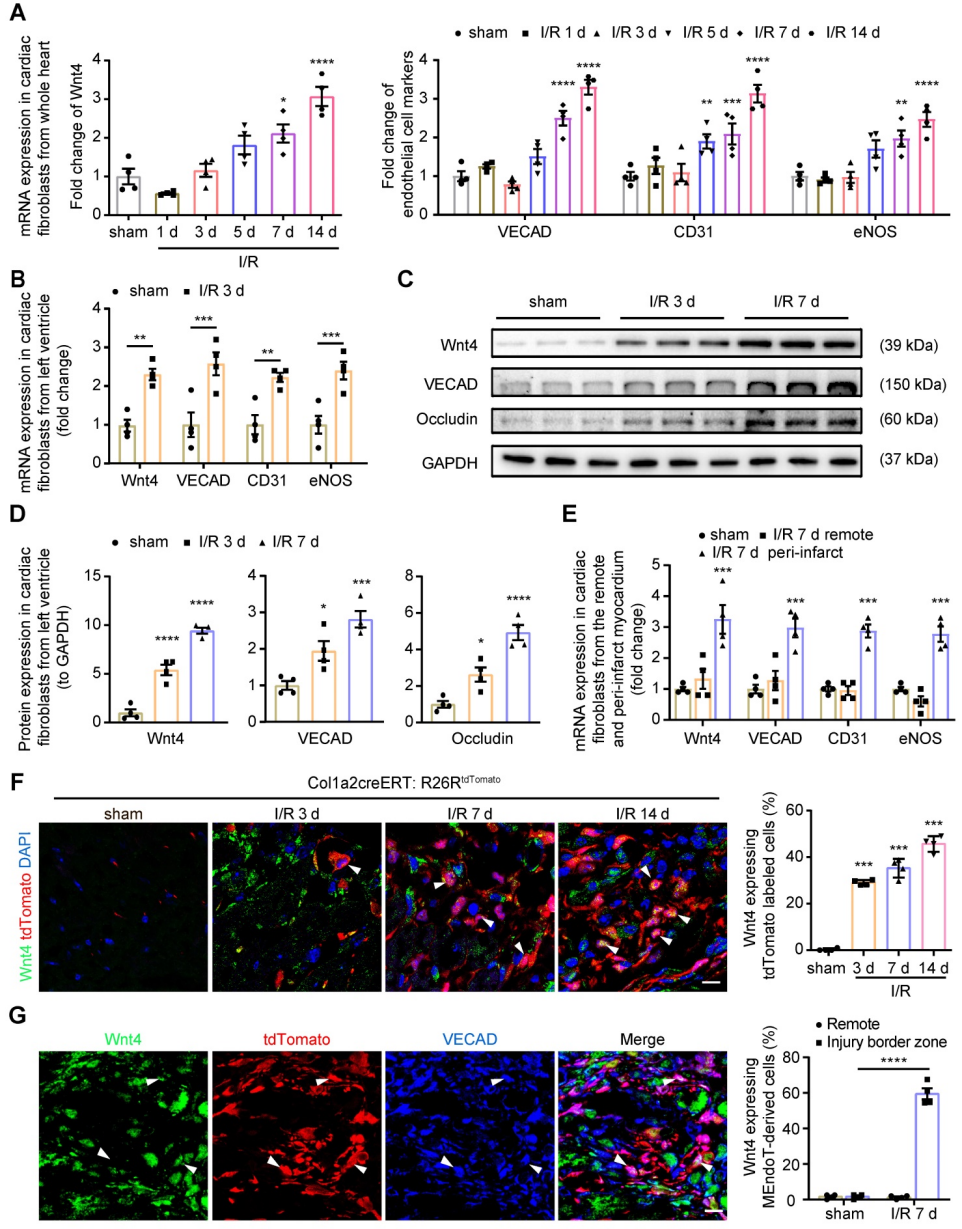
** Wnt4 upregulated in cardiac fibroblasts after acute ischemic reperfusion (I/R) injury.** (**A**) qPCR analysis of Wnt4 and endothelial cell marker genes in cardiac fibroblasts isolated within 24 h from whole heart tissue, n = 4. (**B**) qPCR analysis of Wnt4 and endothelial cell marker genes in cardiac fibroblasts isolated within 24 h from the left ventricular heart tissue early at day 3 post I/R injury, n = 4. (**C-D**) Western blot for Wnt4 and endothelial cell marker genes in cardiac fibroblasts isolated within 24 h from the left ventricular heart tissue post I/R injury and the densitometric quantification, n = 4. (**E**) qPCR analysis of Wnt4 and endothelial cell marker genes in cardiac fibroblasts respectively isolated within 24 h from the remote and peri-infarct myocardium, n = 4. (**F**) Immunofluorescence staining of Wnt4 in injury border zone of heart section from Col1a2-CreERT: R26R^tdTomato^ mice post sham or I/R injury and the quantification, n = 4. (**G**) Triple immunofluorescence staining of tdTomato, Wnt4 and VECAD in injury border zone and the quantification, n = 4. (All graphs show mean ± S.E.M; *p < 0.05, **p < 0.01, ***p < 0.001, ****p < 0.0001, using one-way anova and two-way anova compared with sham. Colocalization of fluorophores is indicated by arrowhead. Scale bar: 10 µm).

In our previous study, we found that Wnt4 mRNA expression was significantly upregulated in the whole heart on day 7 after I/R injury 2. To determine the role of Wnt4 in cardiac repair, we first isolated cardiac fibroblasts from the whole heart for qPCR analysis of Wnt4 expression after cell isolation within 24 h. Both Wnt4 and endothelial cell marker gene expression was significantly upregulated on day 7 and 14 after I/R injury (**Figure 1A**). However, we observed significantly increased expression of Wnt4 and endothelial cell marker genes in cardiac fibroblasts isolated within 24 h from the injured left ventricular tissue instead of the whole heart at day 3 post I/R injury (**Figure 1A-B**). Western blot analysis of cardiac fibroblasts isolated within 24 h from the left ventricular heart tissue confirmed the qPCR results that Wnt4 and endothelial cell marker genes were significantly upregulated in cardiac fibroblasts from the left ventricle at an early time point after I/R injury (**Figure 1C-D**). The qPCR analysis of cardiac fibroblasts at day 7 post I/R injury further showed that both the expression of Wnt4 and endothelial cell marker genes significantly increased in cardiac fibroblasts from peri-infarct myocardium. However, no significant change was observed in the expression of Wnt4 and endothelial cell marker genes in cardiac fibroblasts from remote myocardium (**Figure 1E**).

We subjected Col1a2-CreERT: R26R^tdTomato^ mice to I/R injury and performed immunofluorescence staining of heart sections by using the Wnt4 antibody. We found that Wnt4 was mainly expressed in the injury border zone instead of remote areas (**Figure 1F, Figure S1A-B**). In the injury border zone, 29.3% ± 1.1% of the tdTomato-labeled cells started expressing Wnt4 on day 3 after I/R injury, with Wnt4 expression being detected in an increasing number of tdTomato-labeled cells over time, that is, 42.3% ± 4.3% cells on day 7 and 45.6% ± 4.1% cells on day 14 after injury (**Figure 1F**). Triple immunofluorescence staining of tdTomato, Wnt4, and VECAD showed that 61.1% ± 6.4% of the tdTomato-labeled VECAD-expressing cells in the injury border zone also expressed Wnt4, which further indicated the potential role of Wnt4 in MEndoT (**Figure 1G**). Besides, we also investigated the expression of Wnt4 in other cell types at day 7 post I/R injury. The expression of Wnt4 was upregulated in cardiomyocytes after injury, whereas its expression was not observed both before and after injury in endothelial cells, which expressed CD31 (**Figure S1C-D**).

### Wnt4 was a crucial downstream target gene of p53 during MEndoT

**Figure 2:**
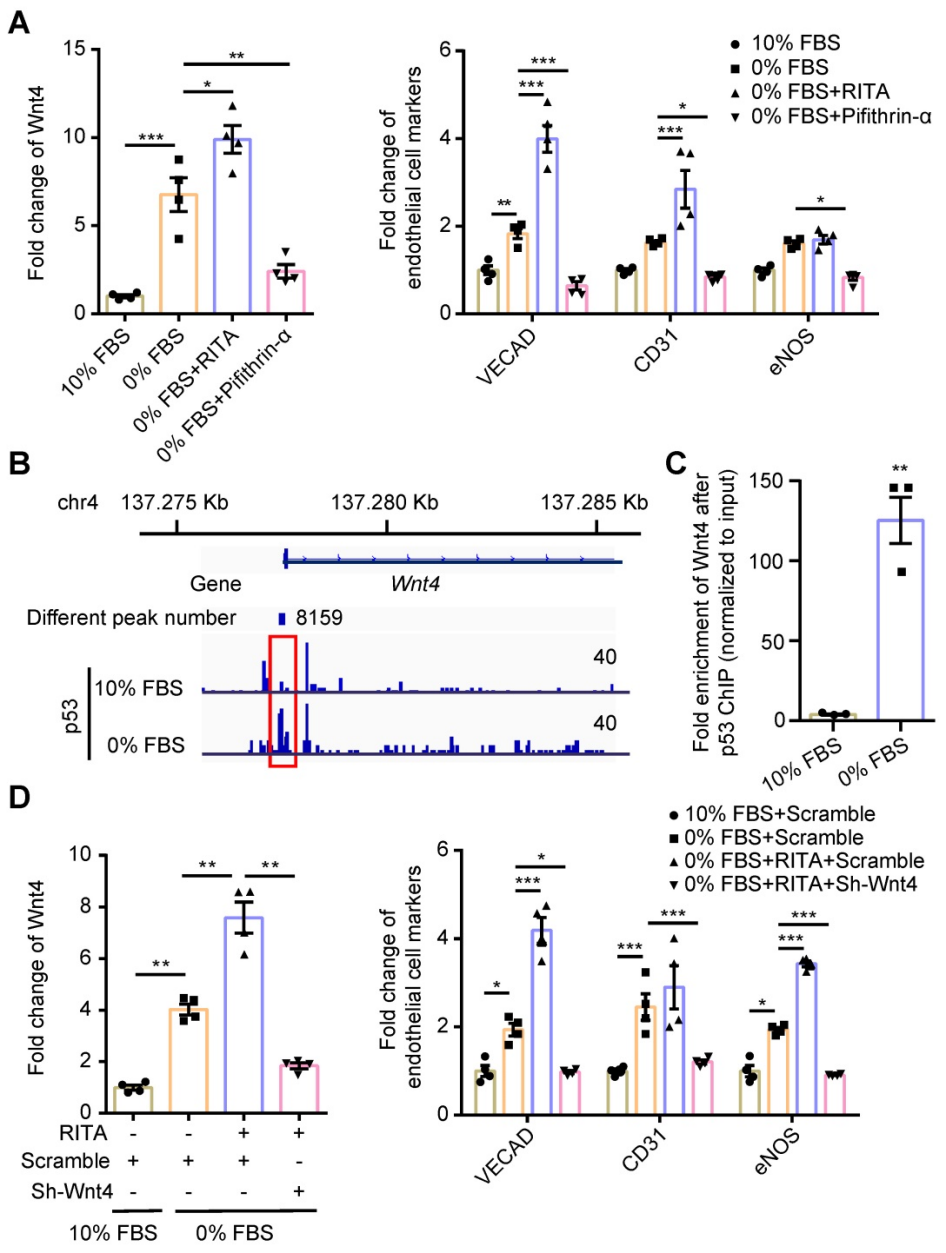
** p53 regulate MEndoT by directly targeting Wnt4 promotor regions.** (**A**) qPCR analysis of Wnt4 and endothelial cell marker genes in cardiac fibroblasts after serum starvation and treated with RITA (p53 activator, 50 µM) and pifithrin-α (p53 inhibitor, 50 µM), n = 4. (**B**) The chromatin immunoprecipitation (ChIP) sequencing for p53 on serum-starved cardiac fibroblasts showed that Wnt4 promoter region was a direct-binding site of p53. (**C**) ChIP-qPCR assay of Wnt4 after p53 ChIP, n = 3. (**D**) The enhanced MEndoT by RITA was inhibited after Wnt4 knockdown, n = 4. (All graphs show mean ± S.E.M; *p < 0.05, **p < 0.01, ***p < 0.001, using unpaired t-test, one-way anova and two-way anova). Note: FBS (fetal bovine serum), RITA (reactivation of p53 and induction of tumour apoptosis).

We have previously shown that serum starvation can induce cardiac fibroblasts undergoing MEndoT in p53-dependent manner 12. We observed that Wnt4 expression was upregulated in cardiac fibroblasts after serum starvation; however, both Wnt4 and endothelial cell marker gene expression could be significantly upregulated or downregulated via modulation of the p53 signaling pathway (**Figure 2A, Figure S2**). These results indicate that Wnt4 is a downstream gene of p53 during MEndoT.

**Figure 3:**
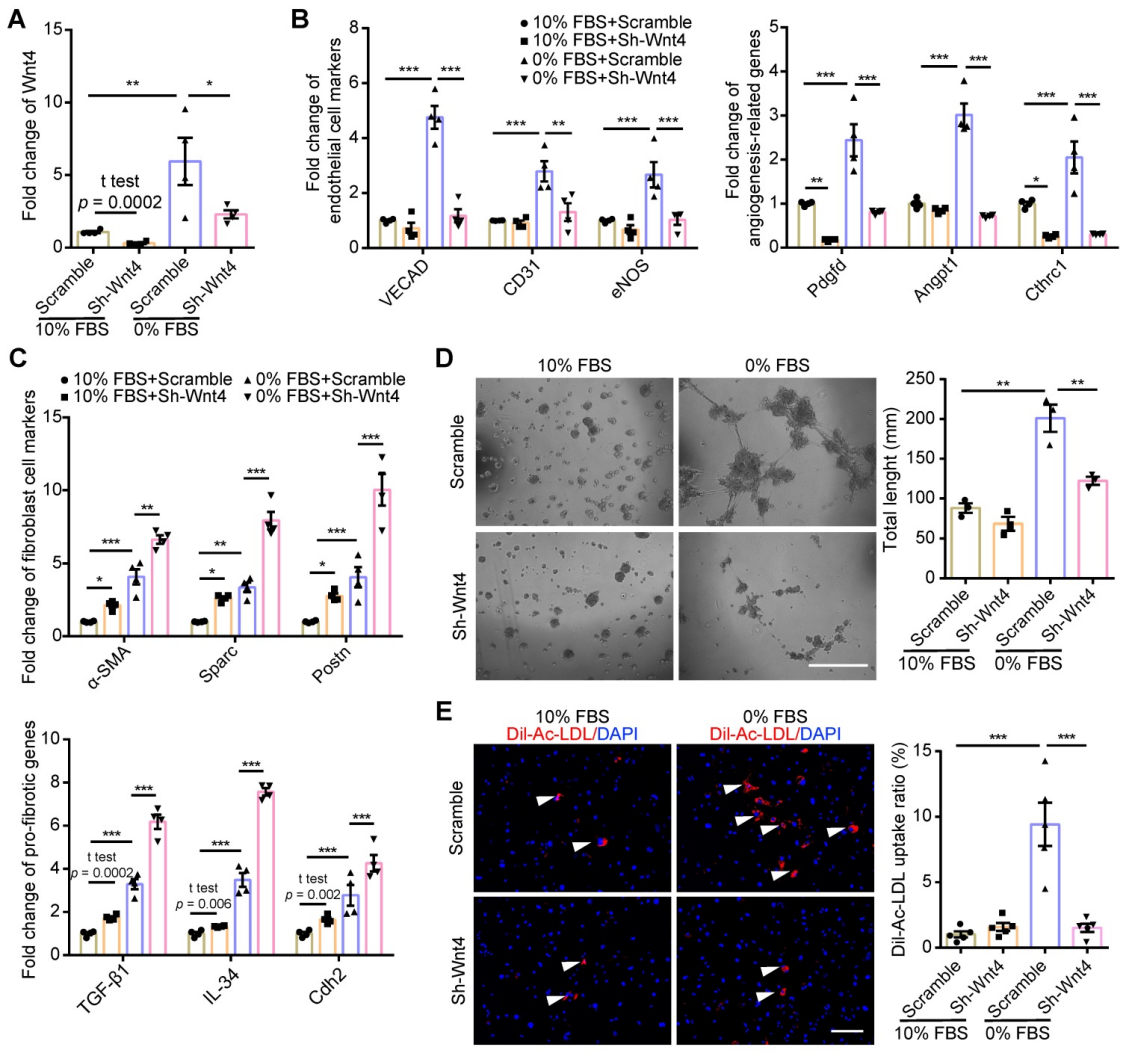
** MEndoT downregulated by Wnt4 knockdown.** (**A-C**) qPCR analysis of gene expression in cardiac fibroblasts after Wnt4 shRNA lentivirus transduction, n = 4. (**A**) Wnt4. (**B**) Endothelial cell marker genes and angiogenesis-related genes. (**C**) Fibroblast cell marker genes and pro-fibrotic genes. (**D**) Capillary tube formation on Matrigel and the quantification, n = 3, Scale bar: 750 µm. (**E**) Ac-LDL intake and the quantification, n = 5, Scale bar: 200 µm. (All graphs show mean ± S.E.M; *p < 0.05, **p < 0.01, ***p < 0.001, using unpaired t-test, one-way anova and two-way anova, fluorophores is indicated by arrowhead.) Note: Angpt1 (angiopoietin 1), α-SMA (actin alpha 2, smooth muscle, aorta), CD31 (Pecam1, platelet and endothelial cell adhesion molecule 1), Cdh2 (cadherin 2), Cthrc1 (collagen triple helix repeat containing 1), eNOS (nitric oxide synthase 3), IL-34 (interleukin 34), Pdgfd (platelet derived growth factor D), Postn (periostin), Sparc (secreted acidic cysteine rich glycoprotein), TGF-β1 (transforming growth factor beta 1), VECAD (cadherin 5).

We performed the ChIP sequencing for p53 by using serum-starved cardiac fibroblasts and found that the Wnt4 promoter region was a direct-binding site for p53 (**Figure 2B**). The ChIP-qPCR assay further confirmed that p53 could directly bind to the promoter regions of the Wnt4 gene when MEndoT was induced in cardiac fibroblasts via serum starvation (**Figure 2B-C**). Furthermore, enhancement of MEndoT via activation of p53 signaling was significantly inhibited when Wnt4 was knocked down (**Figure 2D, Figure 3A, Figure S3A**). Thus, these results demonstrated that Wnt4 was a crucial target gene of p53 for MEndoT regulation.

### Wnt4 was a critical gene for MEndoT induction

**Figure 4:**
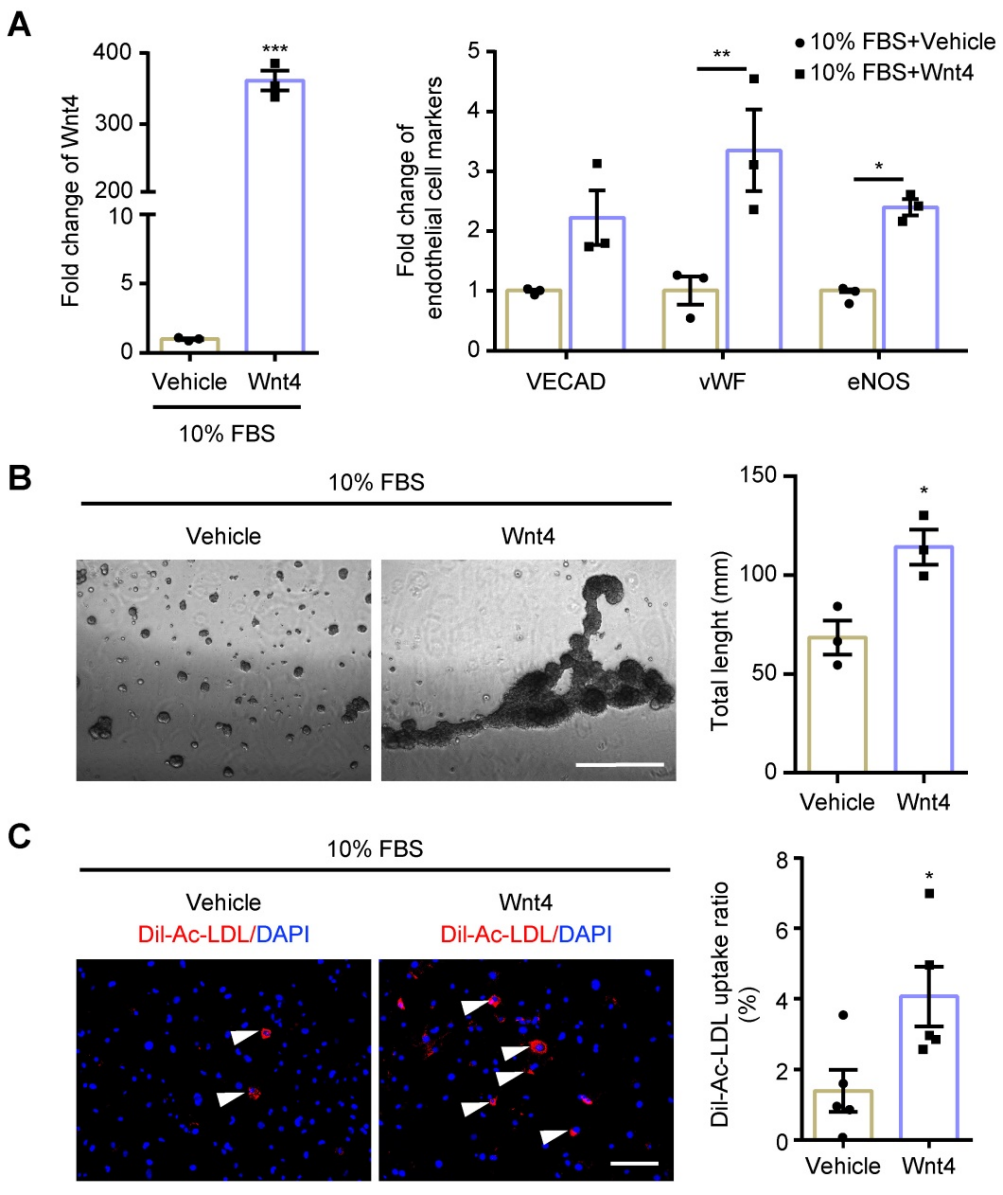
** MEndoT can be induced by Wnt4 overexpression.** The cardiac fibroblasts were transducted with Wnt4 lentivirus and cultured in 10% FBS cell culture conditions. (**A**) qPCR analysis of Wnt4 and endothelial cell markers, n = 3. (**B**) Capillary tube formation on Matrigel and the quantification, n = 3, Scale bar: 750 µm. (**C**) Ac-LDL intake and the quantification, n = 5, Scale bar: 200 µm. (All graphs show mean ± S.E.M; *p < 0.05, **p < 0.01, ***p < 0.001, using unpaired t-test and two-way anova, fluorophores is indicated by arrowhead.)

**Figure 5:**
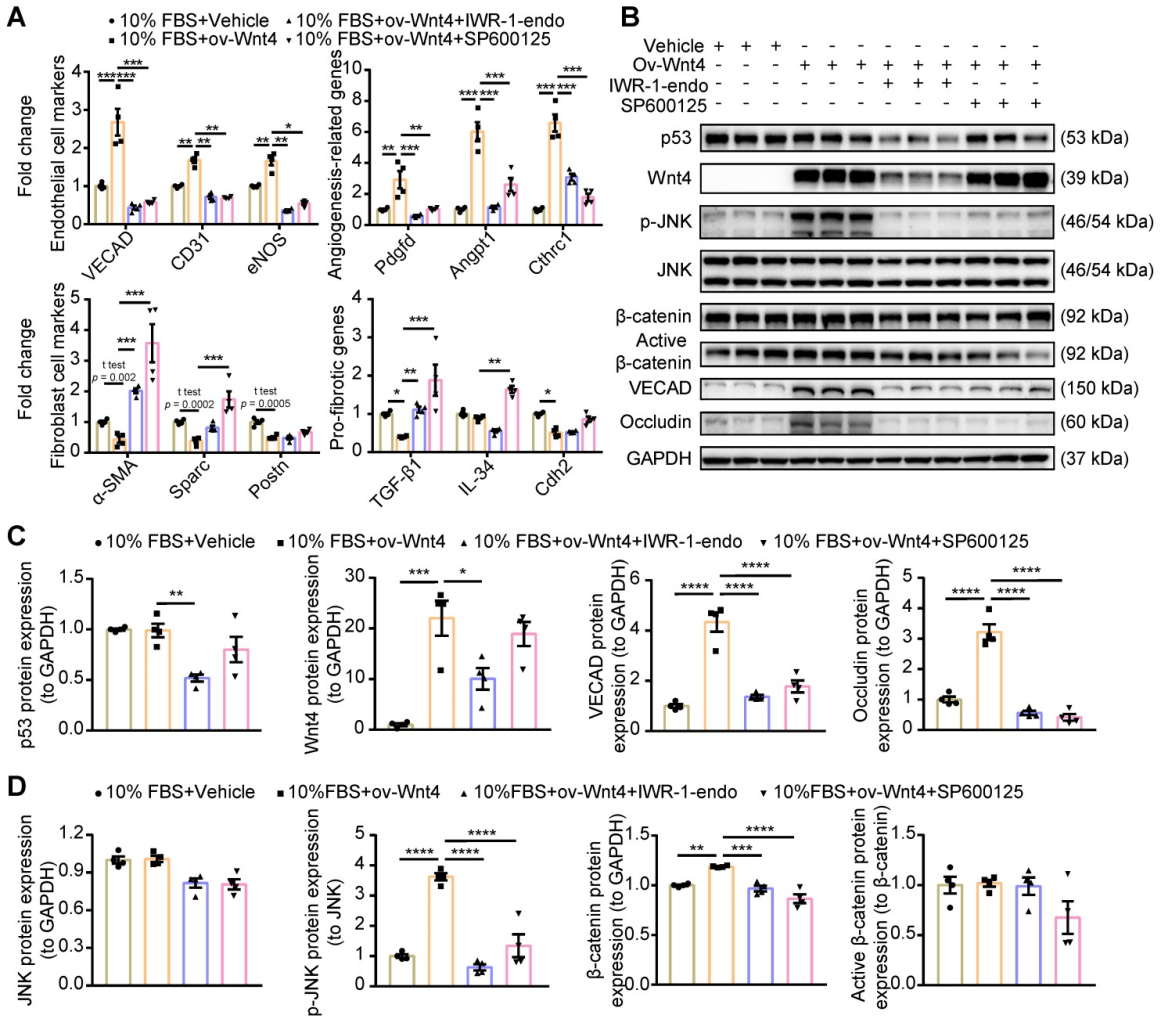
** Wnt4 induce MEndoT via the p-JNK/JNK signaling pathway.** (**A**) qPCR analysis of gene expression after Wnt4 overexpression in cardiac fibroblasts to induce MEndoT and added β-catenin inhibitors IWR-1-endo (50 µM) and p-JNK/JNK pathway inhibitor SP600125 (50 µM). (**B-D**) Western blot for cardiac fibroblasts and the quantification. (All graphs show mean ± S.E.M; *p < 0.05, **p < 0.01, ***p < 0.001, ****p < 0.0001, n = 4, using unpaired t-test, one-way anova and two-way anova.)

Wnt4 in cardiac fibroblasts was knocked down by transduction of shRNA lentivirus. We found that, both in 10% FBS or serum starvation condition, Wnt4 knockdown significantly reduced the expression of endothelial cell marker genes and angiogenesis-related genes, while significantly increased the expression of fibroblast cell marker genes and pro-fibrotic genes (**Figure 3A-C, Figure S3A-B**). Furthermore, Wnt4 knockdown also significantly decreased capillary tube formation on Matrigel by 36.6% (**Figure 3D**) and Ac-LDL intake by 83.9% under serum starvation condition (**Figure 3E**). Conversely, Wnt4 overexpression significantly enhanced the expression of endothelial cell marker genes and angiogenesis-related genes, while significantly decreased the expression of fibroblast cell marker genes and pro-fibrotic genes without serum starvation (**Figure 4A, Figure 5A, Figure S4A**). Under 10% FBS cell culture conditions, tube formation and Ac-LDL intake were also significantly enhanced by 66.9% and 192.1%, respectively (**Figure 4B-C**), after Wnt4 overexpression in cardiac fibroblasts. Thus, these results indicate that Wnt4 itself can induce cardiac fibroblasts to adopt the endothelial phenotype under normal cell culture conditions.

### Wnt4 induced MEndoT via the p-JNK/JNK signaling pathway instead of the β-catenin signaling pathway

Wnt4 is classified as a noncanonical Wnt; however, recent research has shown that it exerts effects via the β-catenin signaling pathway 25,29,31-33,36,39. To clarify which Wnt4-involving signaling pathway regulated MEndoT, we overexpressed Wnt4 in cardiac fibroblasts to induce MEndoT and added p-JNK/JNK and β-catenin inhibitors. qPCR analysis showed that both p-JNK/JNK and β-catenin inhibitors significantly inhibited the occurrence of MEndoT and increased the expression of fibrosis related genes (**Figure 5A, Figure S4A**). The western blot results showed that Wnt4 overexpression significantly induced upregulation of p-JNK and endothelial cell markers such as VECAD and Occludin in cardiac fibroblasts in a dose dependent manner (**Figure 5B-D, Figure S4B**); however, we did not observe a significant increase in JNK and p53 levels (**Figure 5B-D**). Although β-catenin levels significantly increased, active β-catenin levels did not show any obvious change after Wnt4 overexpression in cardiac fibroblasts (**Figure 5D**). β-catenin protein that has not been phosphorylated by glycogen synthase kinase-3 (GSK3) at Ser33, Ser37, and Thr41 is the stabilized form of β-catenin and thus is functionally active in the canonical Wnt signaling pathway 40,41. Furthermore, we observed that the p-JNK/JNK inhibitor inhibited the expression of p-JNK and endothelial cell markers induced by Wnt4 overexpression in cardiac fibroblasts; however, Wnt4 and p53 protein levels were not significant affected (**Figure 5B-D**). The β-catenin inhibitor not only significantly inhibited VECAD, Occludin, p-JNK, and β-catenin expression but also significantly decreased p53 and Wnt4 protein levels (**Figure 5B-D**). Our *in vitro* analyses revealed a new mechanism underlying MEndoT regulation, that, Wnt4 specifically regulates MEndoT via the p-JNK/JNK signaling pathway. However, MEndoT is a β-catenin-dependent process that can be inhibited by β-catenin via its effect on p53 and Wnt4 expression.

### Activation of JNK is crucial for Wnt4 regulating MEndoT

**Figure 6:**
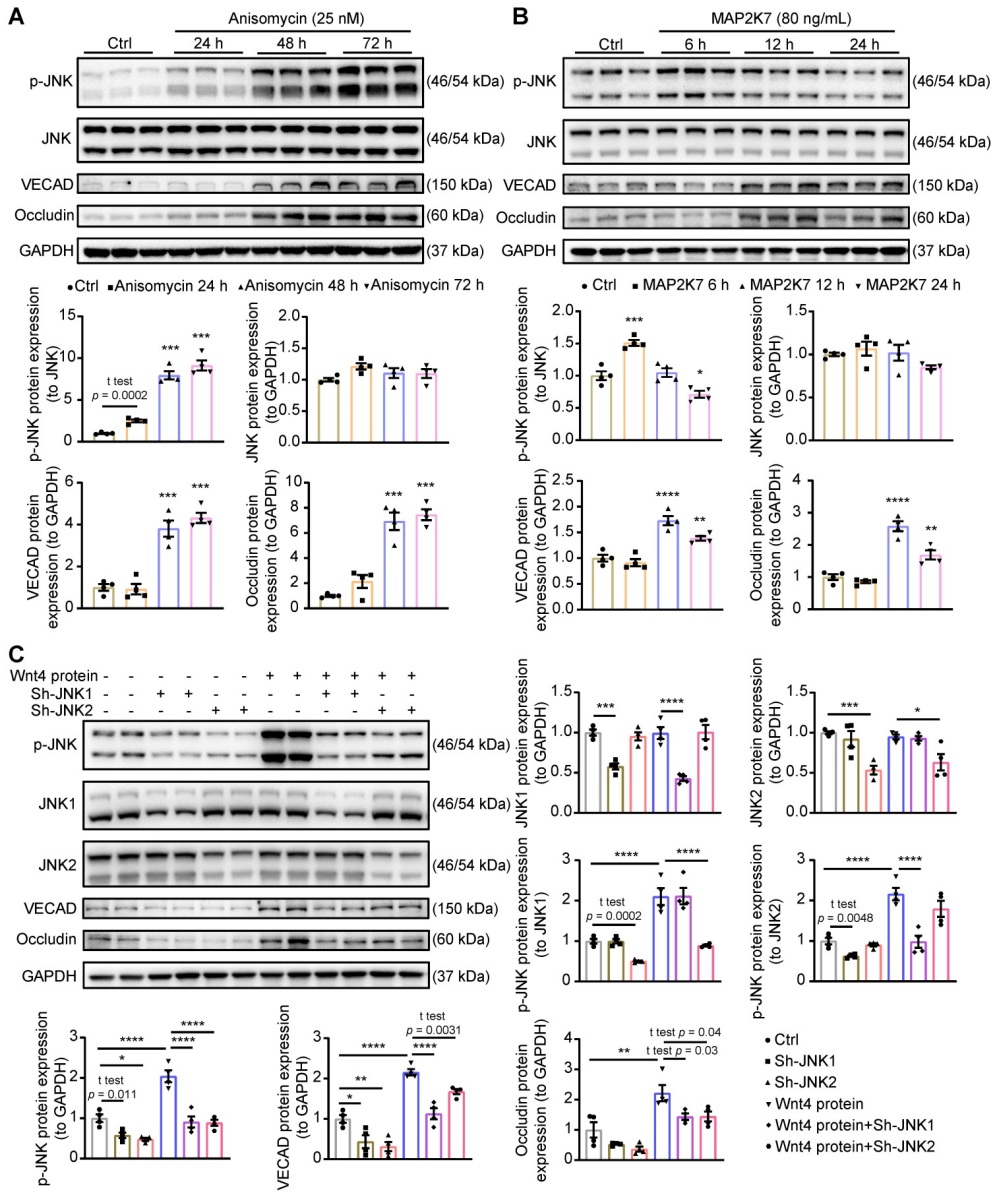
** Activation of JNK is crucial for Wnt4 protein induced MEndoT.** (**A**) Western blot analysis of the expression of p-JNK, JNK, VECAD and Occludin in cardiac fibroblasts after adding with anisomycin (JNK activator, 25 nM) for 24 h, 48 h and 72 h, and the quantification. n = 4. (**B**) Western blot analysis of the expression of p-JNK, JNK, VECAD and Occludin in cardiac fibroblasts after adding with recombinant MAP2K7 (JNK kinase, 80 ng/mL) for 6 h, 12 h and 24 h, and the quantification. n = 4. (**C**) Western blot analysis of the expression of p-JNK, JNK1, JNK2, VECAD and Occludin in cardiac fibroblasts, and the quantification. JNK1 or JNK2 were knock down in cardiac fibroblasts by the transduction of pCLenti-shRNA-virus. MEndoT were induced by adding recombinant Wnt4 (20 ng/mL) for 72 h. n = 4. (All graphs show mean ± S.E.M; *p < 0.05, **p < 0.01, ***p < 0.001, ****p < 0.0001, n = 4, using unpaired t-test and one-way anova.)

Since p-JNK inhibitor which we used is not very specific for JNK, we further investigated the crucial role of p-JNK in the induction of MEndoT. Here, we increased p-JNK level by using pharmocolagical agonist anisomycin 42 or recombinant MAP2K7 43, a kinase specifically activates JNK, in cardiac fibroblasts. Both anisomycin and recombinant MAP2K7 can increase JNK phosphorylation and induce the occurence of MEndoT (**Figure 6A-B, Figure S5A**).

Next, to determine the role of different JNK isoforms in Wnt4-induced MEndoT, we downregulated JNK1 and JNK2 expression in cardiac fibroblasts by shRNAs lentivirus transduction in combination with treatment of human recombinant Wnt4 protein. In this experiment, after treatment with human recombinant Wnt4 protein, p-JNK level was significantly increased, and the expression of endothelial cell markers were upregulated (**Figure 6C, Figure S5B**), which is consistent with the results from Wnt4 lentivirus transduction (**Figure 5B, Figure S4B**). However, knock down of JNK1 or JNK2 attenuated Wnt4-upregulated p-JNK levels, while expression of endothelial markers was also significantly reduced (**Figure 6C**), suggesting that both JNK1 and JNK2 phosphorylation are involved in Wnt4-induced MEndoT.

### Wnt4 upregulation in cardiac fibroblasts was crucial for cardiac function after I/R injury

The pAAV-CGB-DIO-miR30shRNA-WPRE vector containing loxP sites on both sides of an inverted target gene sequence represents a new technology that can help express target genes specifically in Cre-expressing cells (**Figure S6A**) 44. The specifically recombination and expression of target gene in Cre-expressing cells was confirmed by using the pAAV-CGB-DIO-EGFP-Scramble-miR30shRNA-WPRE virus in Col1a2-CreERT: R26R^tdTomato^ mice and the green fluorescent proteins (GFP) was mainly expressed in tdTomato-labeled cardiac fibroblasts (**Figure S6B**). Using this technology, we generated the pAAV-CGB-DIO-Wnt4-miR30shRNA-WPRE virus and administered it to Col1a2-CreERT mice via jugular vein administration. From the next day, tamoxifen was administered via IP injection for 10 days. 5 days after cessation of tamoxifen administration, the Col1a2-CreERT mice were subjected to sham or I/R injury. The pAAV-CGB-DIO-EGFP-Scramble-miR30shRNA-WPRE virus served as the control.

**Figure 7:**
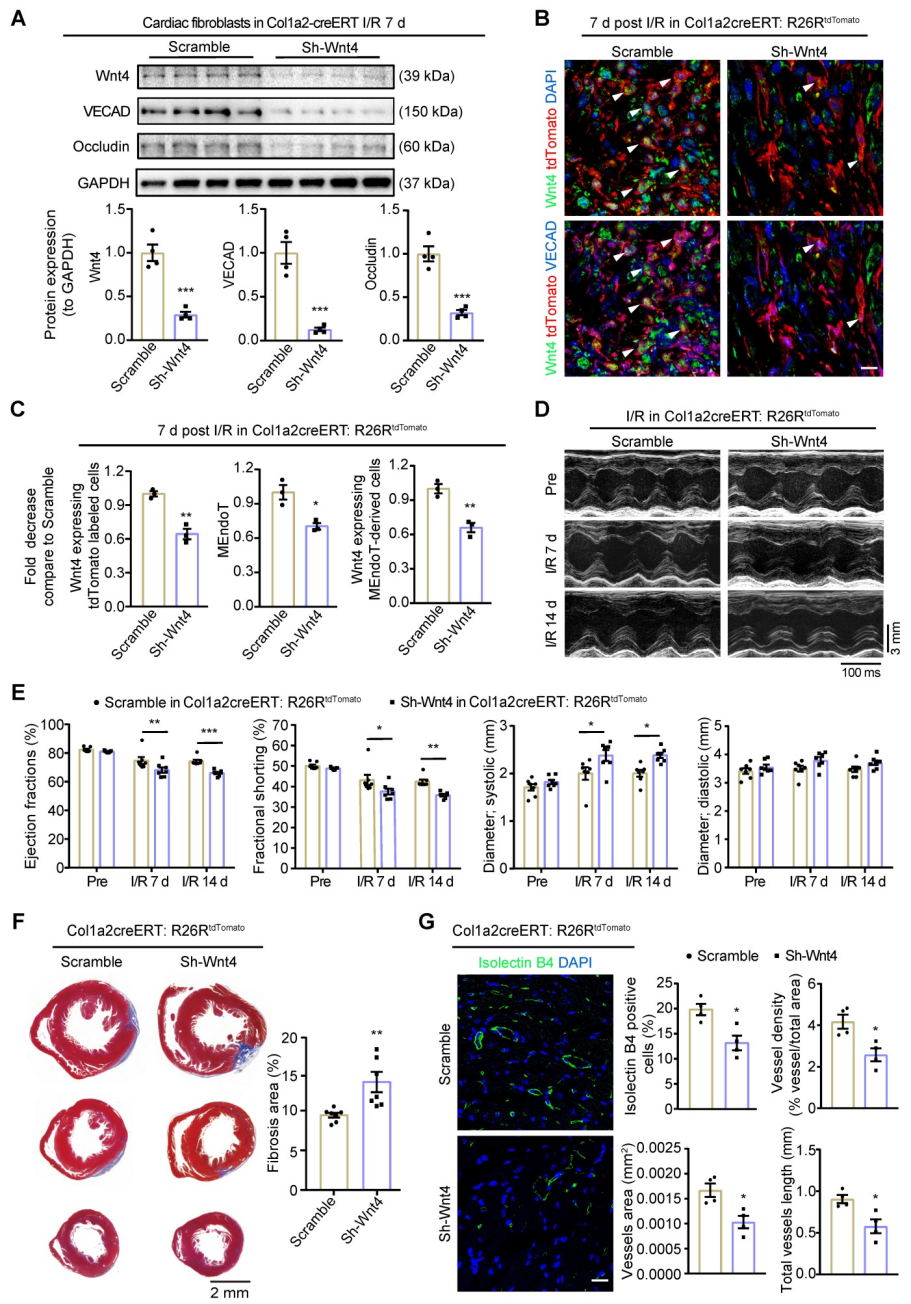
** Wnt4 upregulation in cardiac fibroblasts is crucial for cardiac function after ischemic reperfusion (I/R) injury.** Col1a2-CreERT and Col1a2-CreERT: R26R^tdTomato^ mice subjected to sham or I/R injury after administration of pAAV-CGB-DIO-Wnt4-miR30shRNA-WPRE virus (pAAV-CGB-DIO-EGFP-Scramble-miR30shRNA-WPRE virus serve as scramble control) and tamoxifen. (**A**) Western blot for Wnt4 and endothelial cell marker genes in cardiac fibroblasts isolated within 24 h from whole heart at day 7 post cardiac injury and the densitometric quantification, n = 4 animals/group. (**B-C**) Triple immunofluorescence staining of tdTomato, Wnt4 and VECAD in injury border zone after Wnt4 knockdown in cardiac fibroblasts and the quantification, n = 3 animals/group, Scale bar: 10 µm. (**D**) The representative image of echocardiography M model. (**E**) Cardiac function assayed by echocardiography, n = 7 animals/group. (**F**) Masson' trichrome staining of heart tissue and quantification of fibrosis area by Image J, n = 7 animals/group. (**G**) Immunofluorescence staining of isolectin B4 for vascular density and quantification by Image J and AngioTool, n = 4 animals/group, Scale bar: 20 µm. (All graphs show mean ± S.E.M; *p < 0.05, **p < 0.01, ***p < 0.001, using unpaired t-test and two-way anova, Colocalization of fluorophores is indicated by arrowhead.)

Western blot assays on cardiac fibroblasts isolated from the whole heart within 24 h demonstrated that Wnt4 expression was downregulated by 71% on day 7 after I/R injury after administration of the pAAV-CGB-DIO-Wnt4-miR30shRNA-WPRE virus and tamoxifen (**Figure 7A**). In addition to downregulation of Wnt4 expression, the expression of endothelial cell marker gene VECAD and Occludin were also decreased by 87% and 68%, respectively (**Figure 7A**). By performing immunofluorescence staining on heart sections of Col1a2-CreERT: R26R^tdTomato^ mice on day 7 after I/R injury, we observed that the number of Wnt4-expressing tdTomato-labeled cells decreased by 36% and that of Wnt4-expressing MEndoT-derived cells decreased by 34%, leading to a 30% decrease in MEndoT in the injury border zone (**Figure 7B-C, Figure S6C**). These results indicate that Wnt4 knockdown specifically in cardiac fibroblasts can decrease MEndoT after I/R injury.

The Evans blue and TTC staining results showed that specifically knockdown of Wnt4 in cardiac fibroblasts didn't affect the infarct size 24 h following the I/R injury (**Figure S6D**). However, day 7 and 14 post I/R injury, echocardiography showed that Wnt4 knockdown in cardiac fibroblasts led to worsened cardiac function, increased fibrosis deposition, and decreased vascular density (**Figure 7D-G**). Wnt4 is a secreted protein and may therefore affect endothelial cells; however, considering that Wnt4 was mainly knocked down in cardiac fibroblasts, the decreased vascular density was partly due to the decrease in MEndoT caused by Wnt4-specific knockdown in cardiac fibroblasts (**Figure 7G**). Our findings indicate that Wnt4 expression in cardiac fibroblasts is crucial for cardiac function during I/R injury, which may play a role in MEndoT modulation.

### Wnt4 overexpression in cardiac fibroblasts rescued cardiac function worsened by p53 depletion in cardiac fibroblasts after cardiac I/R injury

**Figure 8:**
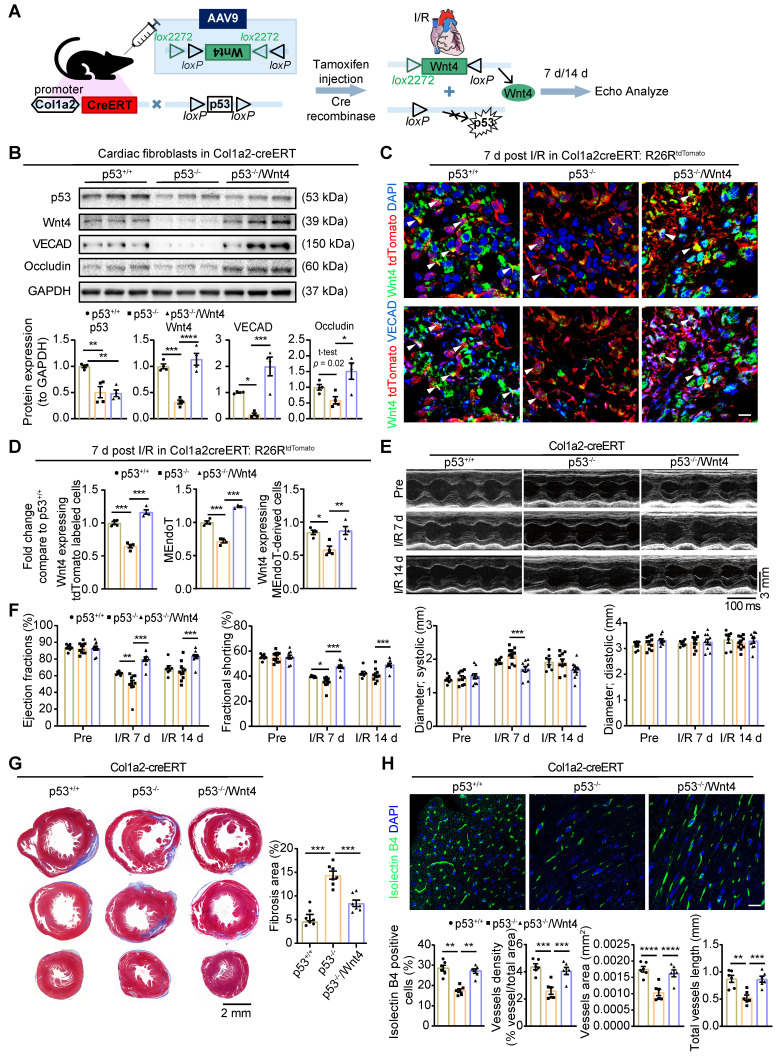
** Wnt4 overexpression in cardiac fibroblasts rescued cardiac function worsened by p53 depletion in cardiac fibroblasts after ischemic reperfusion (I/R) injury.** Col1a2-CreERT: p53 CKO mice subjected to sham or I/R injury after administration of pAAV-CMV-DIO-Wnt4-3xFLAG-WPRE virus (pAAV-CMV-DIO-EGFP-3xFLAG-WPRE virus serve as control) and tamoxifen. (**A**) Schematic diagram show p53 knockdown and Wnt4 overexpression in *Col1a2*^+^ cardiac fibroblasts. (**B**) Western blot for p53, Wnt4 and endothelial cell markers in cardiac fibroblasts isolated within 24 h from whole heart at day 7 post cardiac I/R injury and the densitometric quantification, n = 4 animals/group. (**C-D**) Triple immunofluorescence staining of tdTomato, Wnt4 and VECAD in injury border zone and the quantification, n = 4 animals/group, Scale bar: 10 µm. (**E**) The representative image of echocardiography M model. (**F**) Cardiac function analyzed by echocardiography, p53^+/+^ group n = 7 animals, p53^-/-^ group n = 10 animals, p53^-/-^/Wnt4 group n = 10 animals. (**G**) Masson' trichrome staining of heart tissue and quantification of fibrosis area by Image J, n = 7 animals/group. (H) Immunofluorescence staining of isolectin B4 for vascular density and quantification by Image J and AngioTool, n = 6 animals/group, Scale bar: 20 µm. (All graphs show mean ± S.E.M; *p < 0.05, **p < 0.01, ***p < 0.001, ****p < 0.0001, using one-way anova and two-way anova.). Note: p53^+/+^ is mice with p53-intact, p53^-/-^ is mice with p53 CKO in cardiac fibroblasts, p53^-/-^/Wnt4 is mice with p53 CKO but Wnt4 overexpressed in cardiac fibroblasts.

Our previous research showed that the genetic deletion of p53 in cardiac fibroblasts significantly decreased the induction of endothelial cell gene expression and worsened cardiac function 12. We hypothesized that, if the Wnt4 gene is a crucial target of p53 for regulating MEndoT, overexpression of Wnt4 specifically in cardiac fibroblasts may rescue the cardiac function which was worsened by the genetic deletion of p53 in these cells. Therefore, we generated Col1a2-CreERT: p53CKO mice and administered the pAAV-CMV-DIO-Wnt4-3xFLAG-WPRE virus, which has loxP sites on both sides of an inverted Wnt4 gene sequence (**Figure 8A**), 2 weeks before I/R injury. Tamoxifen was administered the day after the virus was administered. In this mouse model, after tamoxifen administration, Cre-expressing cardiac fibroblasts will show both decreased p53 expression and Wnt4 overexpression in the same cell (**Figure 8A**). The pAAV-CMV-DIO-EGFP-3xFLAG-WPRE virus served as the control. Western blot assays on cardiac fibroblasts isolated within 24 h from the whole heart of Col1a2-CreERT: p53CKO mice show that p53 was significantly downregulated by 49% on day 7 after I/R injury after tamoxifen administration (**Figure 8B**). In addition to downregulated p53 expression, the expression of the endothelial cell marker genes VECAD, Occludin, and Wnt4 decreased by 84%, 41%, and 68%, respectively (**Figure 8B**). However, in Col1a2-CreERT: p53CKO mice administered both the pAAV-CMV-DIO-Wnt4-3xFLAG-WPRE virus and tamoxifen, Wnt4 expression was upregulated, and the endothelial cell marker gene expression was significantly rescued in comparison with that in the Col1a2-CreERT: p53CKO mice. Immunofluorescence staining on heart sections further confirmed the western blot results that the expression of the endothelium-specific genes in cardiac fibroblasts is specifically regulated by the p53 signaling pathway and that Wnt4 is a crucial downstream target gene of p53 for MEndoT regulation (**Figure 8C-D, Figure S7A**).

Based on the TUNEL staining on heart section upon acute 24 h I/R injury, we found specifically knockdown of p53 and Wnt4 overexpression in cardiac fibroblasts didn't affect the apoptosis of cardiomyocyte (**Figure S7B**). However, day 7 and 14 post I/R injury, echocardiography showed that cardiac function worsened, fibrosis deposition increased, and vascular density decreased in mice with p53 knockdown specifically in cardiac fibroblasts (**Figure 8E-H**). Conversely, after wnt4 overexpression in cardiac fibroblasts with p53 depletion, we observed that the worsening cardiac function from p53 CKO mice was rescued (**Figure 8E-H**). The decrease in fibrosis deposition and increase in vascular density may, at least partly, be due to the increase in MEndoT induced by Wnt4 overexpression.

## Discussion

In the current study, we investigated the spatio-temporal expression of Wnt4 and revealed that Wnt4 is a “cardiac response to injury” gene that driving early repair events by regulating MEndoT in the heart. Wnts are dynamically expressed during cardiac development and participate in cardiogenesis; however, the expression of most Wnts in the adult heart is low 15. Using Wnt antagonists such as secreted frizzled-related protein (Sfrp2) to determine the role of Wnts in cardiac repair has led to discrepant results in myocardial outcomes that indicate the complexity of Wnts in regulating cardiac repair 21,22,45,46. In this regard, it is very necessary to determine the pathophysiological role of specific Wnts in cardiac repair. In our previous studies, we screened the expression of 19 Wnts following I/R injury and found that canonical Wnt1 and noncanonical Wnt4 expression were significantly upregulated following cardiac injury. We had earlier focused on the role of Wnt1 and revealed a novel function of Wnt1 as a “cardiac response to injury” gene that aided in driving early repair events in the heart by activating the epicardium and cardiac fibroblasts 2. In contrast to profibrotic role of Wnt1 in cardiac repair, our findings not only support the antifibrotic roles of Wnt4 that are consistent with the findings of some previous studies indicating the potential antifibrotic role of Wnt4 in skin and renal fibrosis 32,33, but also support the proendothelial roles of Wnt4 that are also consistent with some previous studies that mesenchymal stem cells can improve cardiac function after cardiac injury by regulating endothelial cells via the Wnt4 35,36.

MEndoT has been found to be beneficial for cardiac repair and the p53 signaling pathway involves it 12; however, the mechanism underlying MEndoT regulation was unclear. p53 is a multifunctional gene whose activation or overexpression may have a complex effect. As one of crucial target gene of p53 during MEndoT, Wnt4 can induce the occurring of MEndoT even in normal cell culture condition. In contrast to recent studies that Wnt4 exerts its effects via the β-catenin signaling pathway 25,29,31-33,36,39, Wnt4 induces fibroblasts to adopt the endothelial phenotype via the p-JNK/JNK signaling pathway instead of the β-catenin signaling pathway. However, Wnt4 expression is dependent on β-catenin, which indicates that the Wnt4/β-catenin signaling pathway reported 25,29,31-33,36,39 may be attributable to the crosstalk between the canonical and noncanonical pathways during cell fate transformation. MEndoT-derived cells are an endothelial-like highly heterogeneous cell population 10. Improving the functional maturity of MEndoT-derived cells is important for cardiac repair. However, considering it is a complex process involving multiple genes and steps, in-depth analysis on the functional maturity of MEndoT-derived cells has not been focused in the current study. Further research on identification of other crucial genes involved in MEndoT regulation will help to achieve the objective of precisely orchestrating cardiac fibroblasts into a functional endothelial cell fate instead of a myofibroblast fate.

In all, our findings demonstrate an antifibrotic and proendothelial roles of Wnt4 in cardiac repair which not only provide new insight into the mechanism for cardiac repair, but also provide a potential gene to precisely regulate MEndoT for treatment of heart disease.

## Supplementary Material

Supplementary figures.
