# Supplementary material for: Wnt4 is crucial for cardiac repair by regulating mesenchymal-endothelial transition via the phospho-JNK/JNK
Source: Theranostics. 2022 May 13;12(9):4110–26. doi: 10.7150/thno.71392 (PMC9169355; doi:10.7150/thno.71392)
Supplement: Supplementary file 1 — Supplementary figures. [file thnov12p4110s1.pdf]

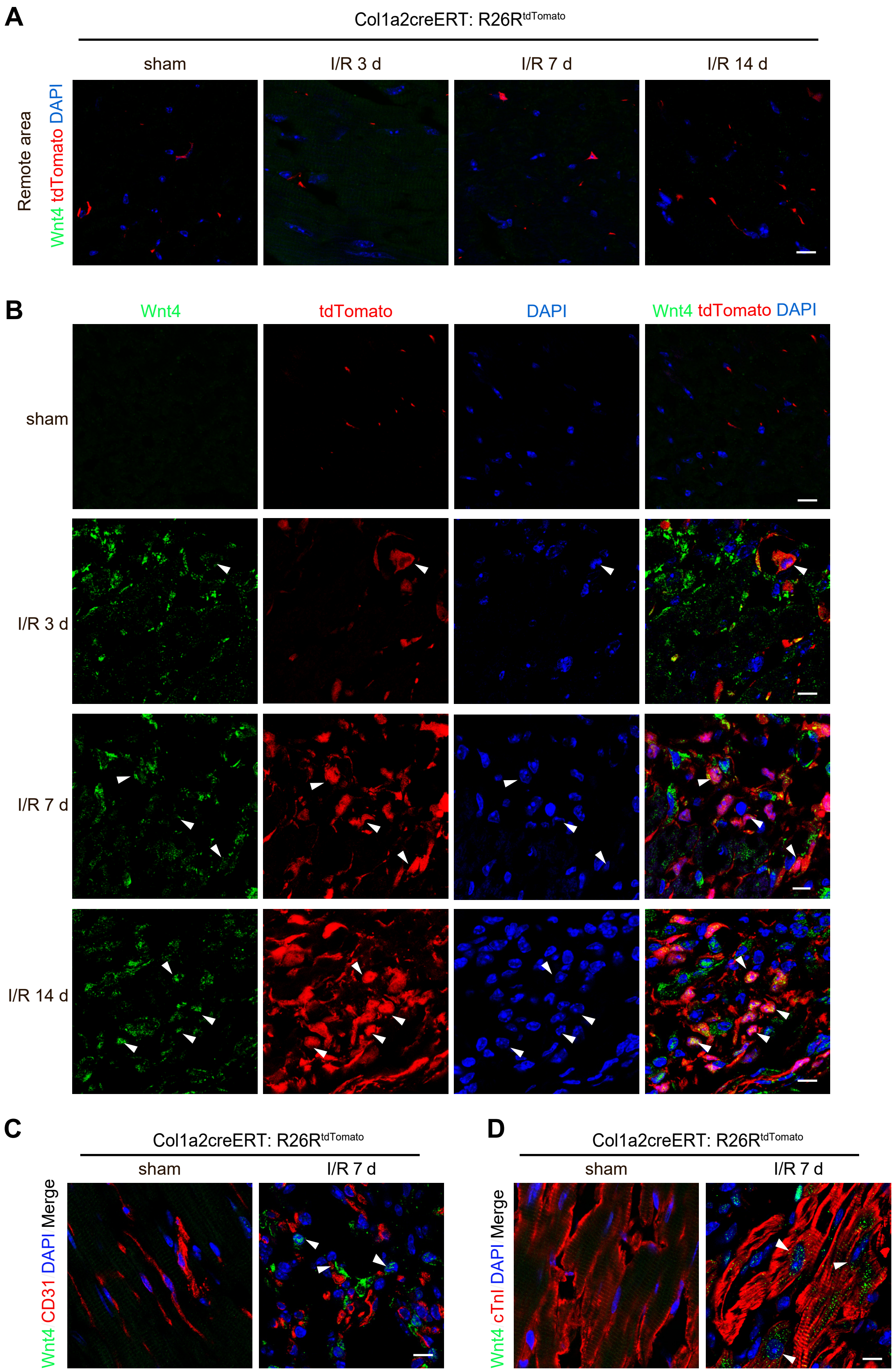

Supplemental Figure 1: Wnt4 was upregulated in cardiac fibroblasts after acute cardiac ischemic reperfusion (I/R) injury

Immunofluorescence staining on heart section of Col1a2-CreERT: R26R^{tdTomato} mice after sham or I/R injury. (**A**) Wnt4 expression in remote area. (**B**) Wnt4 expression in the injury border zone. Single channel fluorescence image of Figure 1F. (**C**) Double immunofluorescence staining of Wnt4 and CD31 in injury border zone. (**D**) Double immunofluorescence staining of Wnt4 and cTnI in injury border zone. (n = 4 animals/group. Colocalization of fluorophores is indicated by arrowhead. Scale bar: 10 μm.)

Figure S2

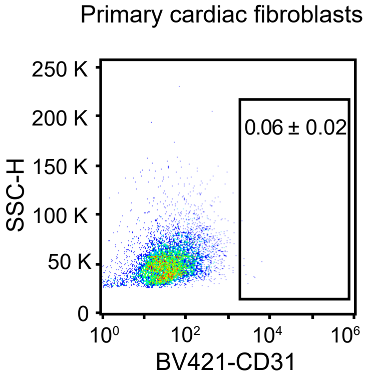

Supplemental Figure 2: Flow cytometric analysis showed that the isolated cardiac fibroblasts did not express endothelial cell marker CD31 (mean ± S.E.M, n = 3)

Figure S3

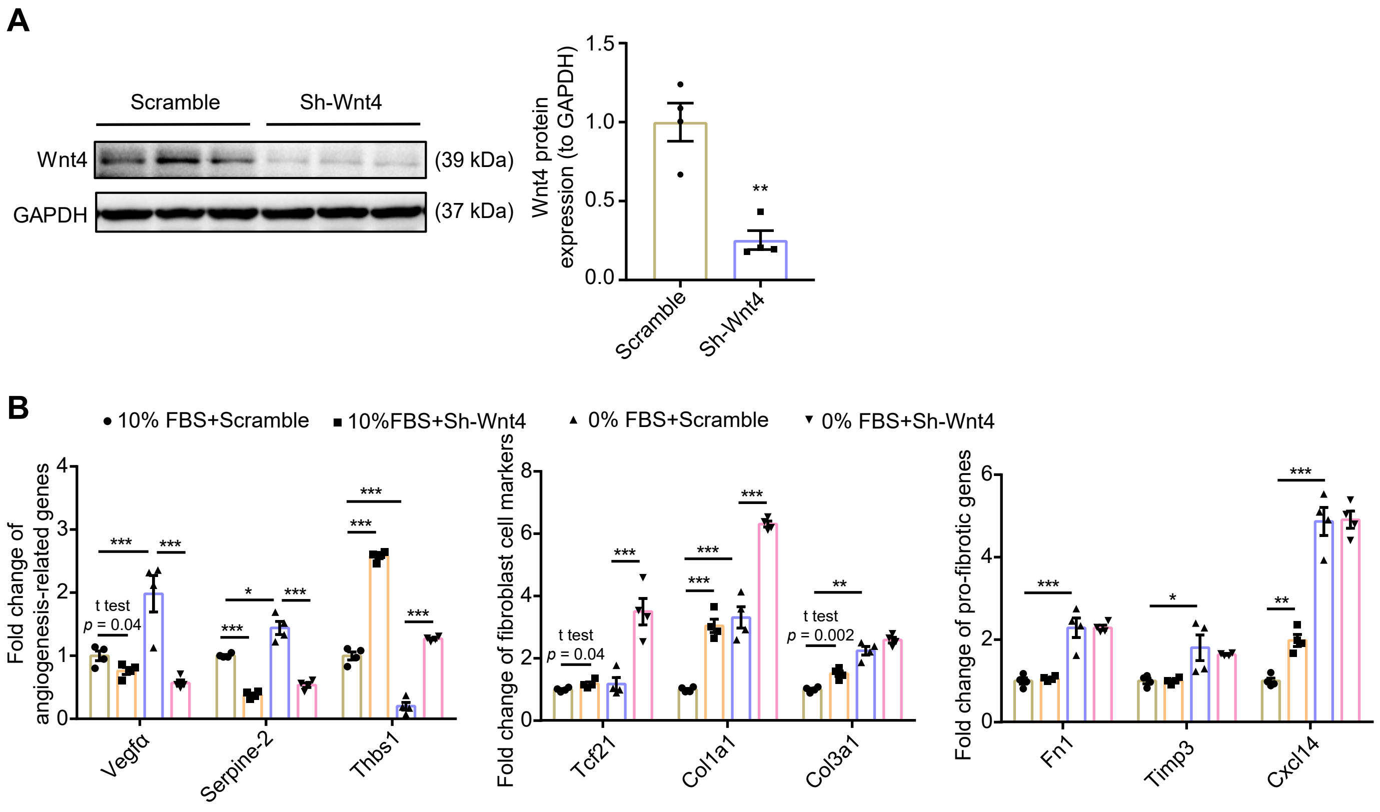

Supplemental Figure 3: Gene expression in cardiac fibroblasts after Wnt4 shRNA lentivirus transduction
(**A**) Western blot assay for the expression of Wnt4 in cardiac fibroblasts at 48 h after Wnt4 shRNA lentivirus transduction (MOI = 80). (**B**) qPCR analysis of gene expression in cardiac fibroblasts both in 10% FBS and serum-starvation. (All graphs show mean ± S.E.M; **p* < 0.05, ***p* < 0.01, ****p* < 0.001, n = 4; using unpaired t-test and two-way anova. Note: Col1a1 (collagen, type **I**, alpha 1), Col3a1 (collagen, type **III**, alpha 1), Cxcl14 (chemokine (C-X-C motif) ligand 14), Fn1 (fibronectin 1), Serpine-2 (serine (or cysteine) peptidase inhibitor, clade E, member 2), Tcf21 (transcription factor 21), Thbs1 (thrombospondin 1, inhibitor of angiogenesis), Timp3 (tissue inhibitor of metalloproteinase 3), Vegfα (vascular endothelial growth factor, alpha).

Figure S4

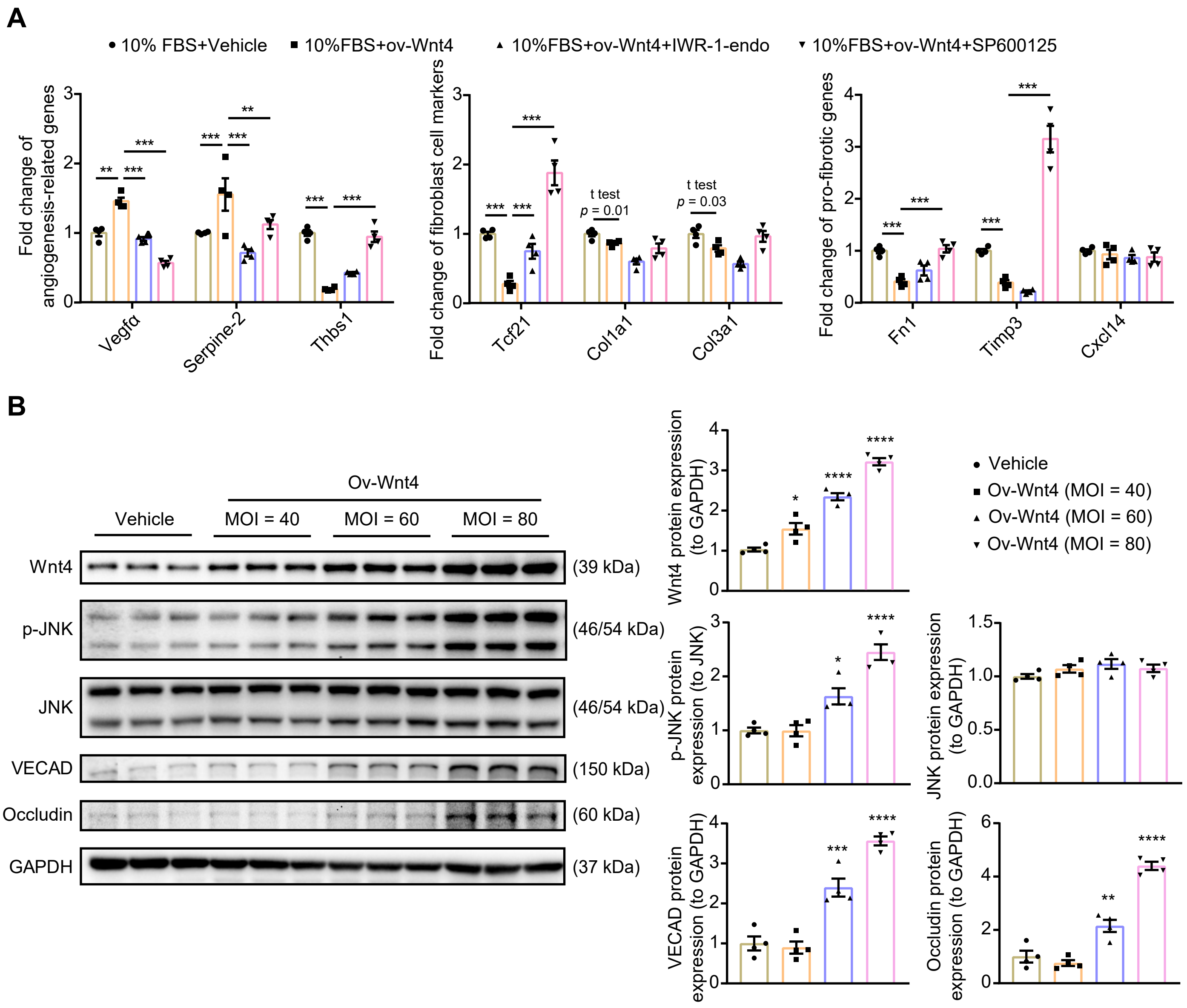

Supplemental Figure 4: Gene expression in cardiac fibroblasts after Wnt4 lentivirus transduction and cultured in 10% FBS cell culture conditions
(**A**) qPCR analysis of gene expression in cardiac fibroblasts after Wnt4 lentivirus transduction followed by 48 h treatment of β-catenin pathway inhibitor IWR-1-endo (50 μM) and p-JNK/JNK pathway inhibitor SP600125 (50 μM), respectively.(**B**) Western blot assay for the expression of Wnt4, p-JNK, JNK, VECAD and Occludin after Wnt4 lentivirus transduction with different MOI (MOI = 40, 60, 80), and the quantification. (All graphs show mean ± S.E.M; **p* < 0.05, ***p* < 0.01, ****p* < 0.001, *****p* < 0.0001, n = 4. using unpaired t-test, one-way anova and two-way anova.)

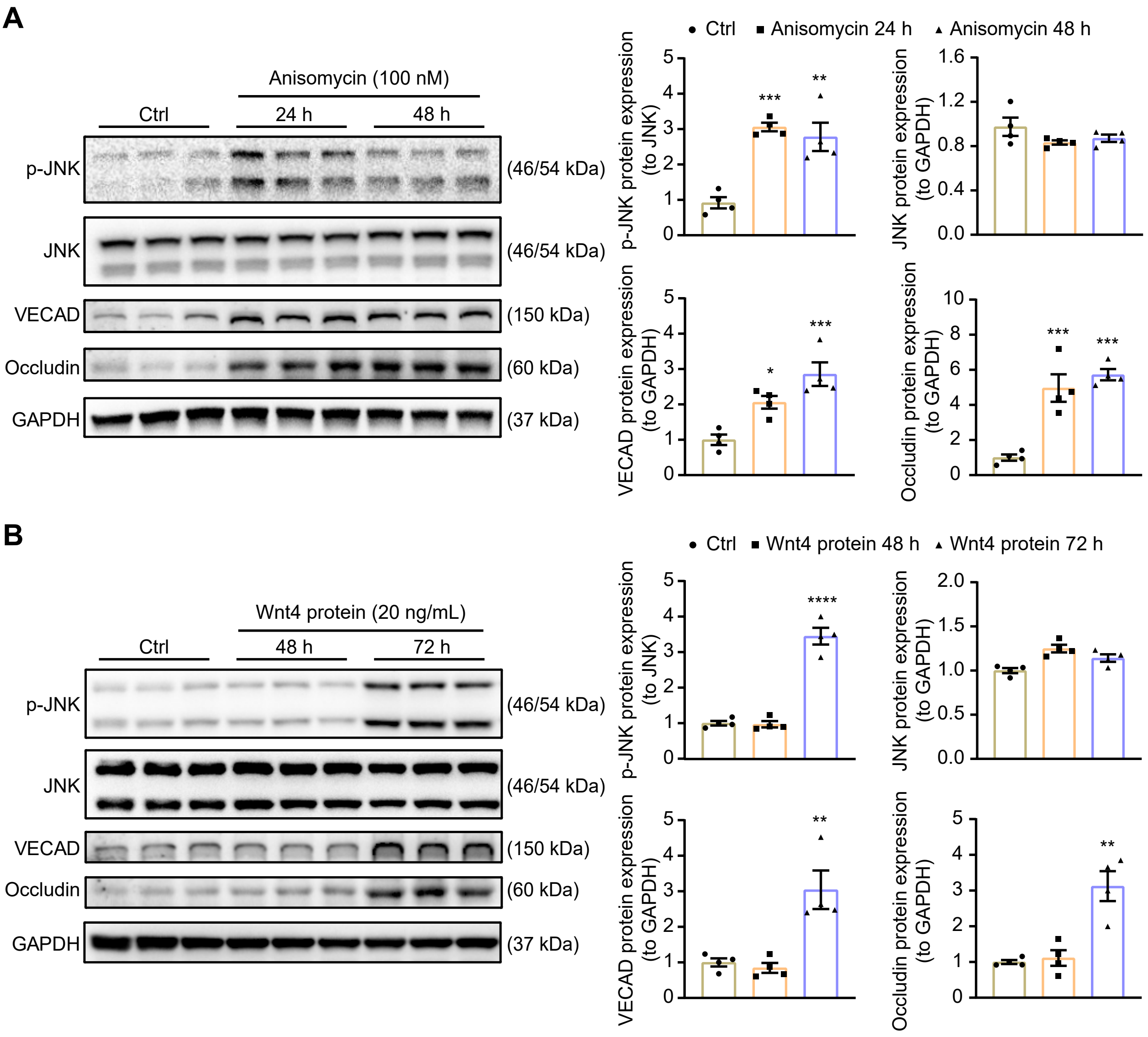

Supplemental Figure 5: Both Wnt4 recombinant protein and activation of p-JNK/JNK signal pathway induce MEndoT

Western blot assay for the expressions of p-JNK, JNK, VECAD and Occludin in cardiac fibroblasts, and the quantification. (**A**) Cardiac fibroblasts were treated with anisomycin (JNK activator, 100 nM) for 24 h and 48 h, respectively. (**B**) Cardiac fibroblasts were treated with recombinant Wnt4 protein (20 ng/mL) for 48 h and 72 h, respectively. (All graphs show mean ± S.E.M; *$p < 0.05$, **$p < 0.01$, ***$p < 0.001$, ****$p < 0.0001$, n = 4, using one-way anova.)

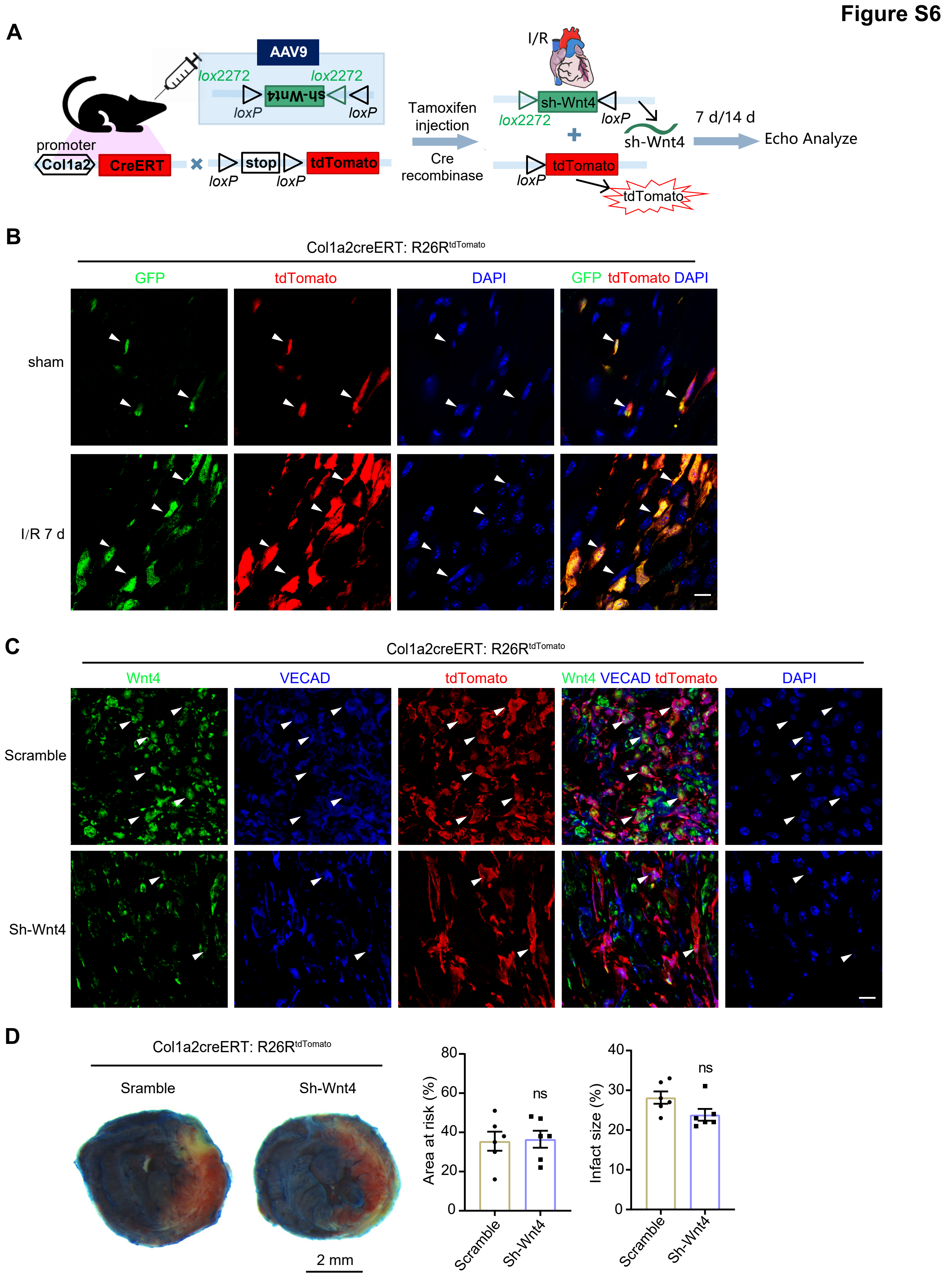

Supplemental Figure 6: Wnt4 upregulation in cardiac fibroblasts is crucial for cardiac function after ischemic reperfusion (I/R) injury

(**A**) Schematic diagram for Wnt4 knockdown in *Col1a2*$^{+}$ cardiac fibroblasts after administration of pAAV-CGB-DIO-Wnt4-miR30shRNA-WPRE virus into Col1a2-CreERT or Col1a2-CreERT: R26^{tdTomato} mice. (**B**) GFP specifically recombinated and expressed in tdTomato-labeled cardiac fibroblasts of Col1a2-CreERT: R26^{tdTomato} mice after administration of pAAV-CGB-DIO-EGFP-Scramble-miR30shRNA-WPRE virus and tamoxifen. (**C**) Single channel fluorescence image of Figure 7B, n = 3 animals/group. (**D**) Evans blue and TTC staining of the heart 24 h after ischaemia-reperfusion injury. The expression of Wnt4 in cardiac fibroblasts was knocked down by administration of pAAV-CGB-DIO-EGFP-Wnt4-miR30shRNA-WPRE virus and tamoxifen in Col1a2-CreERT mice. The infarct area and area at risk analysis were analyzed with Image J software. n = 6 animals/group. (Colocalization of fluorophores is indicated by arrowhead. Scale bar: 10 μm. All graphs show mean ± S.E.M; ns $p > 0.05$, using unpaired t-test.)

Figure S7

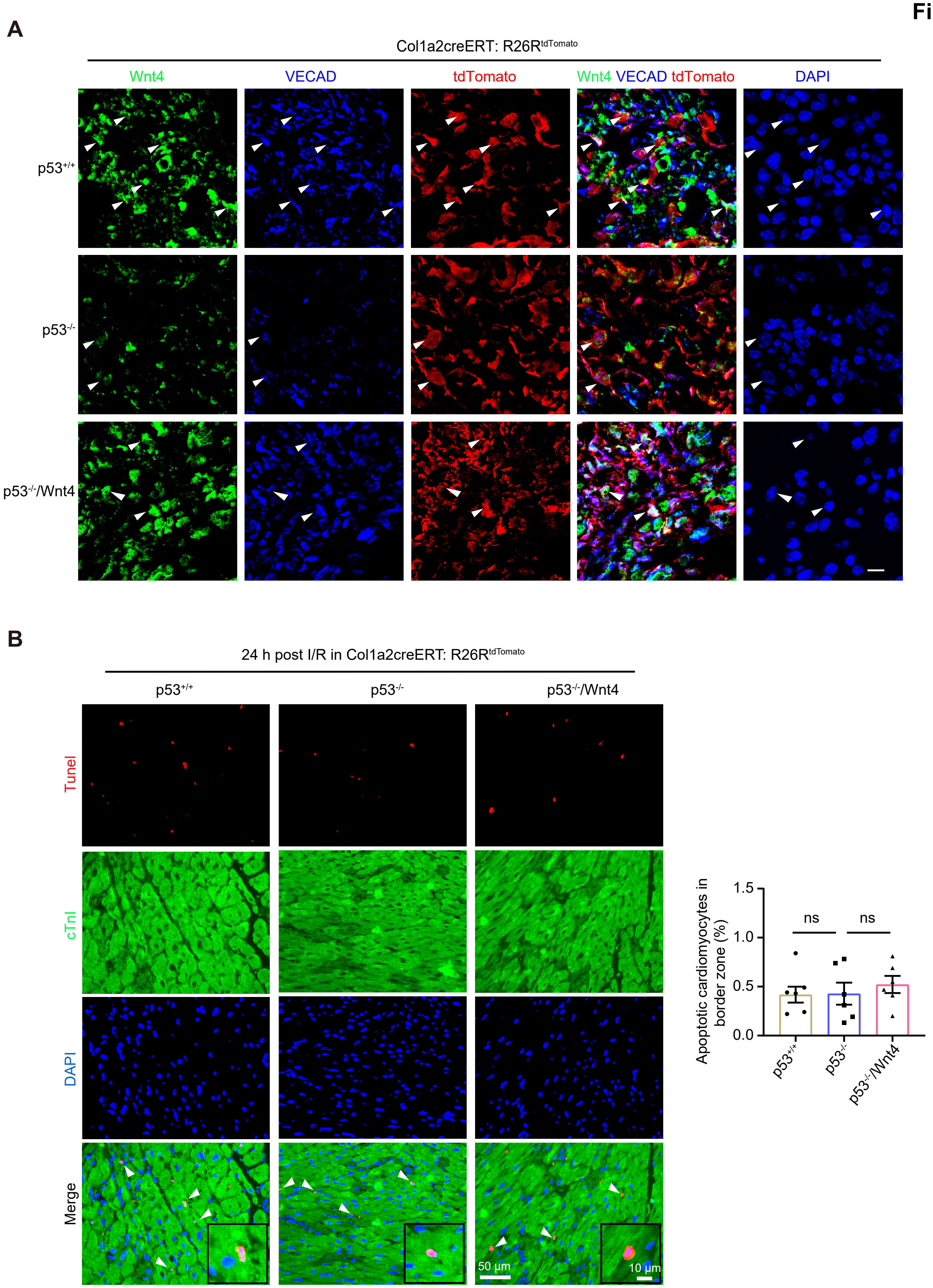

Supplemental Figure 7: p53 knockdown in cardiac fibroblasts didn't affect the cardiomyocyte viability upon acute 24 h ischemic reperfusion (I/R) injury

(**A**) Single channel fluorescence image of Figure 8C. Triple immunofluorescence staining of tdTomato, Wnt4 and VECAD in injury border zone after p53 knockdown and Wnt4 overexpression in cardiac fibroblasts, n = 4 animals/group. $p53^{+/+}$ is mice with p53-intact, $p53^{-/-}$ is mice with p53 CKO in cardiac fibroblasts, $p53^{-/-}$/Wnt4 is mice with p53 CKO but Wnt4 overexpressed in cardiac fibroblasts. Colocalization of fluorophores is indicated by arrow-head. Scale bar: 10 μm. (**B**) Cardiac apoptosis in the myocardium was assessed by TUNEL assay in heart sections from $p53^{+/+}$, $p53^{-/-}$ and $p53^{-/-}$/Wnt4 mice after acute 24 h I/R injury. The white arrowhead indicates apoptotic cells in the sections. n = 6 animals/group. (All graphs show mean ± S.E.M; ns $p > 0.05$, using one-way anova.)
